# Open educational resources: undertheorized research and untapped potential

**DOI:** 10.1007/s11423-020-09907-w

**Published:** 2020-11-30

**Authors:** David A. Wiley

**Affiliations:** Lumen Learning and Brigham Young University, Provo, UT USA

**Keywords:** Open educational resources, Student success, Cost savings, OER-enabled pedagogy

## Abstract

This paper is in response to the manuscript entitled “Open educational resources and college textbook choices: a review of research on efficacy and perceptions” (Hilton in Educ Technol Res Dev 64(4): 573–590, 2016) from a theoretical perspective. The response describes the way many of the papers reviewed by Hilton were undertheorized, limiting their potential for impact. A brief summary of more recent research shows one current direction toward stronger theorization of OER research. Over the short-term, including during the rapid shift to digital learning catalyzed by the COVID-19 pandemic, OER adoption can be expected to save college students money and close the achievement gap between Pell-eligible students and their wealthier peers. Over the longer term, this benefit will likely disappear, and faculty will need to more fully explore the affordances of the 5Rs in order to create dramatic improvements in success for all students.

## Introduction

Hilton (2016) provides a review of nine studies assessing the impact of faculty decisions to adopt open educational resources (OER) on a range of student outcomes. These studies were all conducted in the United States, and this response focuses on OER in the US context.

Informally, OER are teaching, learning, and research materials that can be copied, edited, and shared freely and legally. More formally, Creative Commons (n.d.) defines open educational resources as:

- Teaching, learning, and research materials that are either (a) in the public domain or (b) licensed in a manner that provides everyone with free and perpetual permission to engage in the 5R activities.
  - Retain—make, own, and control a copy of the resource
  - Reuse—use your original, revised, or remixed copy of the resource publicly
  - Revise—edit, adapt, and modify your copy of the resource
  - Remix—combine your original or revised copy of the resource with other existing material to create something new
  - Redistribute—share copies of your original, revised, or remixed copy of the resource with others (para. 2)

Hilton’s review found that outcomes are similar for students whose faculty adopt OER and students whose faculty adopt traditionally copyrighted materials. While a small number of studies found positive or negative effects on student outcomes, the majority found no significant differences.

### Limitations

Many of the articles reviewed in Hilton (2016), including some articles on which I was an author, are woefully undertheorized. They are essentially media comparison studies or, to be more precise, license comparison studies. Without conceptualizing an explanatory mechanism—a reason to believe a difference might exist—they simply compare the outcomes of students whose required course materials are openly licensed with those whose materials are traditionally copyrighted. A stronger theoretical framework, including a hypothesized explanatory mechanism, is required for comparative research to provide useful insights. The reader should not be surprised when reviews of research that lack a sufficient theoretical framework (like many of the articles reviewed by Hilton) find no significant differences.

As a consequence of their copyright licensing, many OER are available to students at no or low cost. This provides a contrast with traditionally copyrighted textbooks, which are frequently incredibly expensive (The Student PIRGS 2018). Therefore, questions about potential differences in student outcomes when faculty adopt OER can sometimes be reframed as questions about the impact of the price of required course materials on student outcomes. If required course materials contribute meaningfully to student outcomes, and if some students are unable to afford access to those materials, there is reason to believe that there may be a gap in student outcomes between those who can afford them and those who cannot.

Wiley (2017) demonstrated that OER integrated into interactive courseware can close this gap. Using eligibility for Pell grants as a proxy for students’ ability to afford their required course materials, Wiley used multiple regression to control for students’ previous academic performance, age, race, gender, enrollment status, and other differences to isolate the effect of Pell eligibility on the final grades of 5622 students at eight institutions in an introductory business course. The analysis also showed that Pell eligible students who used OER integrated into interactive courseware had final grades that were indistinguishable from their wealthier peers, but Pell eligible students using traditionally copyrighted materials or OER outside the courseware context had final grades significantly lower than their wealthier peers.

Colvard, Watson, and Park (2018) studied 21,822 students in eight courses at a single institution, examining the impact of OER adoption on sub-groups of students. In three isolated comparisons of Pell recipients versus non-recipients, non-white students versus white students, and part-time versus full-time students, the authors found that Pell recipients, non-white students, and part-time students each benefited more from their faculty’s decisions to adopt OER than other students.

Grimaldi, Basu Mallick, Waters, and Baraniuk (2019) explored this idea further, naming it the access hypothesis. “The access hypothesis states that OER benefits learning by providing access to critical course materials, and therefore predicts that OER should only benefit students who would not otherwise have access to the materials” (p. 1). Using simulation analysis, they demonstrate that if researchers fail to account for students who would have had access to required course materials even had they been expensive, they will likely be unable to detect any effect of OER adoption on student outcomes. The authors suggest that failure to account for the access hypothesis may contribute to the large number of “no significant difference” findings in the research on OER impacts.

### Application

Adopting OER as part of an emergency shift to digital learning during the COVID-19 pandemic will save students money. When OER are used in accordance with evidence-based teaching practices, they can also help close the achievement gap between lower income and higher income students. But two important points are worth considering when thinking of OER as part of a long-term strategy.

First, while the cost of traditionally copyrighted educational materials has historically been much higher than the price of OER, the cost of textbooks has plateaued for the first time in decades (Perry 2020). As publishers respond to the price pressure created by OER in the course materials market, the difference in the prices of OER and traditionally copyrighted resources is likely to continue to decrease. If the access hypothesis holds, the impact of OER on student outcomes attributable to affordability will decrease in parallel. In other words, adopting OER may not be a long-term strategy for saving students significant amounts of money or closing the achievement gap between lower income and higher income students. If the success of OER programs is measured in terms of cost savings and closing this gap, these programs will likely become less successful over time.

Second, and much more importantly, closing the achievement gap between poorer students and their wealthier peers is not nearly enough. We need to dramatically improve outcomes for all students. For example, only 30% of students graduate from 2 year degree programs within 3 years (National Center for Education Statistics 2019). Dramatically improving this and other student outcomes will require more than adopting cheaper textbooks. Faculty, students, instructional designers, and others will need to think more deeply about using OER together with evidence-based teaching and learning practices, as well as exploring the implications of the 5R affordances in order to find novel, OER-enabled pedagogies (Wiley and Hilton 2018), if they aim to radically improve student outcomes. This may be where the true power and potential of OER lies.

### Future work

Open educational resources are growing in popularity among faculty, students, and administrators (Seamans and Seamans 2018). As their popularity grows, the need to improve our understanding of their potential to impact learning and other measures of student success increases proportionally. If it is to contribute meaningfully to that task, future research on the impact of OER must be grounded in a theoretical framework that provides a clear rationale for why a reasonable person would expect OER use to impact student learning. As researchers move beyond license comparison studies and begin to propose and test concrete explanatory mechanisms for a hypothesized OER effect, our understanding will progress much more rapidly.
